# Effectiveness of an educational module on nurses’ performance regarding safe administration and adverse effects of neuromuscular blocking agents in critically ill patients

**DOI:** 10.1186/s12912-025-03600-0

**Published:** 2025-07-22

**Authors:** Shimaa Attia Ali, Enas Ebrahiem Elsayed, Ayman Muhammad Kamel Senosy

**Affiliations:** 1 Adult Health Nursing Department-Faculty of Nursing, Helwan University, Cairo, Egypt; 2 Lecturer of Medical – Surgical Nursing Department, Faculty of Nursing, Ain Shams University, Cairo, Egypt

**Keywords:** Educational module, Neuromuscular blocking agents, Nurses’ performance, Administration, Adverse effects

## Abstract

**Background:**

Inadequate nurse’s knowledge and poor skills regarding neuromuscular blocking agents lead to harm administration and associated with adverse effects and negative outcomes. Addressing these deficiencies through targeted education and training programs is essential for enhancing patient safety and improving healthcare outcomes.

**Aim:**

to assess effectiveness of an educational module on nurses’ performance regarding safe administration and adverse effects of neuromuscular blocking agents in critically ill patients.

**Design:**

A quasi-experimental design was used.

**Setting:**

The study was conducted at intensive care units affiliated to Medical Ain-Shams university hospital.

**Subjects:**

A purposive sample of nurses (80) was working in previously mentioned setting.

**Data collection tools:**

Nurses’ knowledge questionnaire and nurses’ practices observational checklist, and nurses reported adverse effect.

**Results:**

the present study revealed that, there were improvement of the studied nurses’ satisfactory level of knowledge regarding administration of neuromuscular blocking agents, clinical practice guideline recommendation, the studied nurses’ competent level of practice, and reported adverse effect from (11.6, 8.4, 6.3, 8.3, and 7.5%) at pre-implementation to (88.4, 91.6, 93.7, 91.7 and 97.5%) at post-implementation phase, respectively, with a highly statistically significant differences between pre/post at *P* < 0.001.

**Conclusion:**

The implementing educational module had positive large effect size on nurses’ performance (Knowledge and practice) regarding safe administration, recognize and manage the potential adverse effects of neuromuscular blocking agents in critically ill patients throughout pre/post phases of the study at η2 = 0. 729 and 0.833.

**Recommendations:**

Study the effectiveness of simulation, virtual reality, and AI-based tools in improving NMBA-related nursing competencies.

**Clinical trial number:**

Not applicable.

**Supplementary Information:**

The online version contains supplementary material available at 10.1186/s12912-025-03600-0.

## Introduction

Neuromuscular blocking agents (NMBAs) are a class of medications commonly used in anesthesia. NMBAs are used to facilitate airway management, improve surgical conditions, and ensure immobility during critical points in an operation. The NMBAs are administered over 100 million times annually to facilitate tracheal intubation “Thilen et al., [1]”. The neuromuscular junction (NMJ) is a synaptic connection between the terminal end of a motor nerve and a muscle (skeletal/ smooth/ cardiac). It is the site for the transmission of action potential from nerve to the muscle. It is also a site for many diseases and a site of action for many pharmacological drugs “Jimsheleishvili et al., [2]”.

Proprioceptive and kinaesthetic awareness, dynamic stability, preparation and reactionary muscle characteristics, and conscious and unconscious functional motor patterns are four essential components for restoring neuromuscular control and functional stability. The two main types of neuromuscular blocking agents are depolarizing NMBAs (like succinylcholine) and non-depolarizing NMBAs (like atracurium, cisatracurium, rocuronium, and vecuronium). The ideal NMBAs are thought to be a non-depolarizing agent with a quick onset of action, short duration of effect, and few side effects. Non-depolarizing NMBAs, like atracurium, cisatracurium, rocuronium, and vecuronium, are commonly used in clinical practice “Adeyinka & Layer [3]”.

To produce prolonged muscle relaxation, NMBAs work by competitively blocking acetylcholine receptors at the neuromuscular junction. The stereoisomer of atracurium is cisatracurium, and both are benzylisoquinoline NMBAs. Compared to atracurium, cisatracurium has four times the potency on NMBAs. Atracurium and cisatracurium are the preferred drugs for continuous infusion “Cook & Simons [4]”.

The latter, cisatracurium, is the NMBA of choice in the treatment of critically ill patients requiring NMBAs as it is unrelated to histamine release. Rocuronium and vecuronium are both steroid NMBAs. Rocuronium has a rapid start and intermediate duration of action, making it suited for rapid sequence intubation. Comparatively speaking, vecuronium acts similarly to rocunorium in terms of duration but with a slower onset “Tsolaki et al., [5]”.

Neuromuscular blocking agents are frequently used to facilitate endotracheal intubation, to provide surgical relaxation, and to facilitate controlled mechanical ventilation in both the operating room and the intensive care unit. NMBAs have no sedative, hypnotic, or analgesic side effects, but NMBAs may indirectly decrease metabolic demand, prevent shivering, decrease nonsynchronous ventilation, decrease Inter cranial pressure, and improve chest wall compliance. The use of these agents are obviously linked with long-term ventilation and NMBAs exacerbate muscle immobilization; accumulation may also have a direct toxic effect on skeletal muscle, or increase its susceptibility to corticosteroid-mediated muscle weakness. NMBAs should be used only when necessary and if a continuous infusion is deemed necessary, agents whose metabolism is independent of renal and hepatic function should be selected to reduce the chance of accumulation “Taylor [6]”.

The risk of NMBA-related adverse events (AEs) was deferent according to the system organ class (SOC). The top five SOCs with the highest number of AEs among the four NMBAs were general disorders and administration site conditions, respiratory, thoracic and mediastinal disorders, cardiac disorders, injury, poisoning and procedural complications and immune system disorders “Jiang et al., [7]”. In the aspect of cardiac disorders, all four NMBAs exhibited medium signals (+ 2). In terms of immune system disorders, the signals of rocuronium were identified as strong signals (+ 3), atracurium and cisatracurium were medium signals (+ 2), while vecuronium showed a weak signal (+ 1). For “pregnancy, puerperium and perinatal conditions,” atracurium showed a weak signal (+ 1), while the other three NMBAs showed no signal. For hepatobiliary disorders, cisatracurium showed a weak signal (+ 1) “Li et al., [8]”.

Numerous medications interact with and intensify the effects of NMBAs. This has clinical implications for cyclosporin, lithium, inhalational antibiotics, and cosmetics. Polymyxin and calcium channel blockers might make it difficult to reverse the block. But other medications, such lithium, carbamazepine, and phenytoin, can make you resistant to NMBAs. Additionally, the onset of succinylcholine is prolonged by precurarization using a non-depolarizing medication. If succinylcholine is taken while recovering from pancuronium blockade or after neostigmine for reversal, its effects are noticeably prolonged. Plasma cholinesterase hydrolyses succinylcholine, and medications that inhibit its activity, such as organophosphate, cyclophosphamide, echothiopate, and contraceptive pills, may result in a prolonged block “Cook & Simons [4]”.

For nurses knowledge regarding clinical practice guidelines for the sustained NMBA in the adult critically ill patient, there were strong recommendation (Scheduled eye care with lubrication and eyelid closure), weak recommendation (Continuous infusion rather than intermittent boluses, avoid use in status asthmatics, trial of NMBA in life-threatening situations with hypoxemia, respiratory acidosis, and hemodynamic compromise, manage overt shivering in therapeutic hypothermia, peripheral nerve stimulator (PNS) clinical assessment may be a useful tool for determining the depth of blockade, PNS should not be used alone in patients receiving a continuous infusion of NMBAs, implementation of a structured physiotherapy regimen, target blood glucose level < 180 mg/dL, dose NMBA based on ideal body weight) “Renew et al.; Iavarone et al., [9, 10]”.

And also, good practice recommendation (PNS can be used in patients undergoing therapeutic hypothermia, protocols should be utilized to guide NMBA administration in patients undergoing therapeutic hypothermia, analgesic and sedative drugs should be used before and during NMBA, implement measures to reduce risk of unintended extubation, reduce dosing in patients with myasthenia gravis based on PNS use, discontinue NMBAs prior to determining brain death) “Renew et al.; Iavarone et al., [11, 12]”.

Nurses had special consideration regarding the use of NMBAs in acute care settings, which is vitally important. The assessment before NMBAs protect patients from tracheal damage, mortality, and stomach aspiration while also greatly increasing first pass success rates. Though each has unique dangers and requires customized diagnosis and planned interventions, succinylcholine and rocuronium are frequently utilized during emergent intubations. Using a video laryngoscope and making sure overweight individuals receive the right dosage can improve first pass success “Blauvelt et al., [13]”.

The selected NMBA for planned, non-emergent intubations with prolonged paralysis can be tailored to the patient’s requirements. To prevent histamine release, this includes dosing based on acetylcholine up- or down-regulation as well as administration speed. When administering certain NMBAs to high-risk patient populations (coronary artery disease, hyperkalaemia, renal failure, liver failure, and traumatic brain injuries), nurses must take extra precautions. Lastly, careful evaluations are necessary to avoid difficulties due to the vast spectrum of negative effects associated with these medications Blauvelt et al., [13]”.

One of the most recent developments in the educational system that allows nurses to actively engage in the learning process is the educational module. An educational module is a learning resource that has predetermined goals. These goals include teaching learning materials that are necessary for understanding subjects, using various media, and providing learners with specific steps to follow, from goals to evaluation. Additionally, nurses can actively participate in the learning process according to the educational module “Deep et al.; King et al., [14, 15]”.

The educational module is comprised of title, overview, target nurses, educational objectives (knowledge and practice), pre-test, learning activities, and post-test. The evaluation phase is a methodical and ongoing procedure to ascertain whether the educational module has been completed, nurses have met the requirements, and they have communicated the anticipated benefits and level of care “Oshi et al.; Dayalal et al., [16, 17]”.

### Significance

Nurses are the largest group of healthcare workers on the front line of efforts to provide high quality of care. Critical care nurses must have a thorough awareness of the significance of using NMBAs, as well as an appreciation for the elements that influence patient selection, agent selection, dose titration, and side effects, to reduce patient risks and promote the safe and efficient administration. Also, obviously noticed that most nurses are unaware of the negative consequences of NMBAs. Additionally, there hasn’t been any research done on these topics in nursing, or perhaps there hasn’t been any research at all. Because nurses are crucial in managing these health issues, it is vital to assess the impact of educational modules concerning nurses’ knowledge practice, and guidelines for NMBAs administration, adverse effects with patients receiving NMBAs and patients’ clinical outcomes “Elmaboud et al., [18]”.

The administration of neuromuscular blocking agents (NMBAs) presents nursing care challenges such as managing the dangers of extended paralysis, monitoring for consequences such as critical illness myopathy, and maintaining enough sedation and analgesia to avoid patient awareness and discomfort. The goal of research on patient discomfort during intubation and mechanical ventilation (MV) is to lessen suffering brought on by the underlying illness, the critical care setting, and the endotracheal tube “Renew et al. [9]”.

### Aim of the study

This study was conducted with the aim of assessing the effectiveness of educational modules on nurses’ performance regarding safe administration and adverse effects of neuromuscular blocking agents in critically ill patients, through the following:

1. To demonstrate knowledge of nurses regarding the safe administration and practices guidelines for neuromuscular blocking agents in critically ill patients.
2. To understand the role of nursing in monitoring critically ill patients receiving neuromuscular blocking agents.
3. To recognize the potential adverse effects of neuromuscular blocking agents in critically ill patients.
4. To Evaluate the effectiveness of educational module on nurses’ performance regarding safe administration and manage adverse effects of neuromuscular blocking agents in critically ill patients.

### Research hypothesis

#### H1

Nurses who receive the educational module will demonstrate a statistically significant improvement in knowledge scores regarding safe administration of neuromuscular blocking agents compared to pre-intervention scores.

#### H2

The nurses studied will have a statistically significant improvement in the practice level regarding recognizing and managing the potential adverse effects of neuromuscular blocking agents in critically ill patients.

### Operational definitions

#### The educational module

To enhance the quality of care given after implementation, a set of pre-planned objectives (knowledge and practice) for the safe administration practices for neuromuscular blocking agents in critically ill patients and target nurses is designed, including a pre-test, learning materials, and educational activities, followed by a post-test to enhance nurses’ knowledge and practice level.

 It was administered through combination methods that were using a traditional lecture with displaying video-based learning and doing clinical practice, which involved observational checklists to evaluate the nurses’ post-interventions.

#### Research design

Quasi-experimental research design was used to conduct this study. An experiment is a study in which the researcher operates the level of some independent variable and then measures the result. Experiments are potent methods for assessing cause-and-effect relations “Gopalan, Rosinger& Ahn [19]”.

### Technical design

The technical design includes research settings, subjects and tools for data collection.

#### Setting

The study was conducted at (cardiac care unit (25 nurses), medical care unit 1 (15 nurse), medical care unit 2 (15 nurses), chest care unit (10 nurses), blood disease intensive care unit (5 nurses) and neurological care unit (10)) affiliated to Medical Ain-Shams university.

#### Subjects

A purposive sample comprised of (80) staff nurses (total population sampling) who met the inclusion criteria, working at previous mentioned setting affiliated to Medical Ain-Shams university hospital and agree to participate in the study “Crossman [20]”.

#### Inclusion criteria

staff nurses who are.

- Willing to participate in the study.
- Available at the time of study.
- Has experience in delivering healthcare awareness to patients and participated in workshops before.
- Working previously with patients had received neuromuscular blocking agents.

### Tools for data collection

Data for this study were collected using the following tools:

#### Tool I: Nurse structured interviewing questionnaire

An Arabic questionnaire, based on recent literature was developed by the researcher, it included the following parts:

#### Part I: Demographic characteristics of nurses

under study as regards age, gender, marital status, level of education and position, experience year, working hours and training courses.

#### Part II: Nurses knowledge questionnaire

as regards to assess nurses’ knowledge safe administration and adverse effects of neuromuscular blocking agents in critically ill patients (pre/post). It included two main sections.

#### Section I: Nurses’ knowledge regarding Neuromuscular Blocking Agents (pre/post)

This questionnaire was designed by researchers in Arabic language after reviewing the related literature. The items on this sheet were adapted from “Adeyinka & Layer; Iavarone et al. [12–21]”, it included 16 items (Definition neuromuscular blockers: generic and brand names, the steps of neuromuscular junction, the elements of neuromuscular control and function stability, the non-depolarizing and depolarizing neuromuscular blockers agents, therapeutic action, indications, the clinical pharmacokinetics, contraindications and cautions, adverse effects, drug interactions, nursing considerations, nursing assessment for patient before administration, nursing diagnoses, implementation with rationale, and evaluation of patients outcome).

#### Scoring system

The total score of nurses’ knowledge was 16 questions equal 32 grade; (2) grade was given to the correct answer and (1) to the incorrect answer. Total score was considered as the following:

- ≥ 85% was considered satisfactory level of knowledge (*≥* 27.2 grades correct answer).
- < 85% was considered unsatisfactory level of knowledge (< 27.2 grades correct answer).

#### Section II: Nurses’ knowledge regarding clinical practice guidelines recommendation for the sustained neuromuscular blockers agents in the adult critically ill patient (pre/post)

The items on this sheet were adopted from “Murray et al. [22]”, it included 3 mean clinical practice recommendations (Strong recommendation (1 items), weak recommendation (9 items good practice recommendation (6 items)).

#### Scoring system

The total score of nurses’ knowledge was 32 questions equal 64 grade; (2) grade was given to the correct answer and (1) to the incorrect answer. Total score was considered as the following:

- ≥ 85% was considered satisfactory level of knowledge (*≥* 57.4 grades correct answer).
- < 85% was considered unsatisfactory level of knowledge (< 57.4 grades correct answer).

#### Tool II: Nurses’ practical observational checklists

It were adopted from (Bittner; Wiegand [23, 24]”, to evaluate the nurses’ practical competency level throughout administration of neuromuscular blocking agents in critically ill patients: Use, agent selection, administration, and adverse effects. The observational checklists were composed of five main Aspects: (Obtain and document a baseline assessment (8 items), provide sedation/analgesia as ordered (1 items), assessment parameters during neuromuscular blockade (8 items), and necessary interventions during neuromuscular blockade dimension (9 items), and discontinuation of neuromuscular blockade therapy dimension (4 items).

#### Scoring system

The total score of practice observational checklists was 30 questions equal to 60 grades; (2) grade was given to the step which was done correctly and (1) to the step which was done incorrectly or not done. Total score was considered as the following:

- ≥ 85% was considered a competent level of practices (≥ 51 correct actions).
- < 85% was considered incompetent level of practices (< 51 correct actions).

#### Tool III: Nurses’ reported adverse effect regarding administration of neuromuscular blocking agents in critically ill patients

This questionnaire was designed by the researchers in Arabic language after reviewing the related literature to assess adverse effect regarding administration of neuromuscular blocking agents in critically ill patients and adapted from “Li et al., [8]”, it included 20 adverse effect (Anaphylactic reaction, anaphylactic shock, feeling dizzy, burning and pain at injection site, hypotension, bradycardia, tachycardia, arrhythmias, cardiac arrest, hyperthermia, elevation in serum potassium levels, erythema, inadequate respiratory function, bronchospasm, hypoxia, oxygen saturation decreased, muscle weakness, seizure, decreased drug effect, and prolonged drug effect).

#### Scoring system

The total score of nurses’ knowledge was 20 questions equal to 40 grades; (1) grade was given to occur and (2) to Not occur. Total score was considered as the following:

- ≥ 85% was considered Not occur (*≥* 34 grades correct answer).
- < 85% was considered Occur (< 34 grades correct answer).

#### Development of educational module

An educational module was developed for the staff nurses. It was prepared based on analysis of pre-test knowledge scores, review of literature and discussion with the guide and other experts, chose of the checklist, preparation of the first draft and apply content validity of educational module then preparation of the final draft. The educational module is divided into four chapters.

#### Chapter I

It included knowledge regarding neuromuscular blockers agents (Definition neuromuscular blockers: generic and brand names, the steps of neuromuscular junction, the elements of neuromuscular control and function stability, the non-depolarizing and depolarizing neuromuscular blockers agents, therapeutic action, indications, the clinical pharmacokinetics, contraindications and cautions, adverse effects, and drug interactions). The most common used frequently of NMBAs are succinylcholine and rocuronium. And atracurium, vecuronium and cisatracurium are also used, particularly for longer procedures. Added to that the Neuromuscular blocking agents (NMBAs) come usually in two forms: nondepolarizing neuromuscular blocking agents (e.g., vecuronium, cisatracurium, atracurium, mivacurium, rocuronium), and depolarizing neuromuscular blocking agents (e.g., succinylcholine). Also, it includes the sedative agents that are commonly used with NMBAs where the neuromuscular blocking agents (NMBAs) and sedatives are commonly used together, especially the sedative-hypnotic induction agent and used particularly during rapid sequence intubation (RSI) to manage agitation and provide comfort during mechanical ventilation. By rapidly rendering a patient asleep and paralyzed with a sedative and an NMBA, RSI helps regulate airways and reduces unintended physiological reactions during intubation. The most common examples of the co-administered sedatives used with NMBAs include midazolam, propofol, and opioids like morphine. Ketamine can also be used as an adjunct to provide deeper sedation “Adeyinka & Layer [21]”.

#### Chapter II

It included knowledge regarding (Nursing considerations, nursing assessment for patients before administration, nursing diagnoses, implementation with rationale, evaluation of patients’ outcome, and clinical practice guidelines recommendation for the sustained neuromuscular blockers agents in the adult critically ill patient).

#### Chapter III

It included nurses’ practical neuromuscular blocking agents’ administration checklist to assess use, agent selection, administration, and adverse effects. The observational checklists were composed of five main Aspects (Obtain and document a baseline assessment, provide sedation/analgesia as ordered, assessment parameters during administration of neuromuscular blocking agents, necessary interventions during administration of neuromuscular blocking agents, and discontinuation of administration of neuromuscular blocking agents therapy).

#### Chapter IV

It included expected adverse effect regarding administration of neuromuscular blocking agents in critically ill patients (Anaphylactic reaction, anaphylactic shock, feeling dizzy, burning and pain at injection site, hypotension, bradycardia, tachycardia, arrhythmias, cardiac arrest, hyperthermia, elevation in serum potassium levels, erythema, inadequate respiratory function, bronchospasm, hypoxia, oxygen saturation decreased, muscle weakness, seizure, decreased drug effect, and prolonged drug effect).

#### Operational design

The operational design includes preparatory phase, pilot study and field work.

### Preparatory phase

It included reviews of current and past available literature and theoretical knowledge of various aspects of the study using booklets, articles, internet, periodicals and magazines to develop data collection tools.

### Content validity

Content validity was conducted to test representative of all aspects of the study. To produce valid results, the content of a test and measurement method must cover all relevant parts of the topic it aims to be appropriate, relevant, correct, and clear “Middleton [25]”. The juries were five experts, from one medical-surgical nursing professor at the faculty of nursing, Helwan University, and four critical nursing care professors at the faculty of nursing, Ain-shams University. Their opinions were expressed about the format of the tool, the layout, the consistency and the scoring system.

### Testing reliability

Reliability can be estimated by comparing different versions of the same measurement “Middleton [25]”. It tested by using Cronbach alpha test the reliability scores of study tools including Arabic version; the Cronbach alpha for internal consistency of nurse’ knowledge questionnaire, practical observational checklists, and reported adverse effect were (0.903, 0.899 & 0.901) respectively.

### Pilot study

A pilot study was applied to a group of 8 nurses (10% of the sample) to test the applicability of the tools and clarity of the designed questionnaire, as well as to estimate the time needed to answer them. Nurses included in the pilot study were also included in the main study topics, as there were no changes in the tools.

### Field work

This study was conducted through three phases: Assessment phase, educational module implementation phase (Theoretical & practical stage) and evaluation phase:

### I- Implementation phase

- This phase started by selecting nurses before educational module implementation who met the inclusion criteria and explaining basically the aim and nature of the study as well as taking their approval to participate in the study prior to data collection.
- **The structured interview questionnaire** included demographic characteristics and the nurses’ Knowledge questionnaires were completed by the nurses. It took around 30 min to fill it out for each nurse.
- Nurses were observed by the researcher using practical observational checklists to assess their appraisal form, measuring the effectiveness of nursing staff in safe administration and monitoring adverse effects of neuromuscular blocking agents. This method involves noting down what researchers see and what the nurses say about what is happening. It took every nurse a half-hour.
- Based on nurses’ knowledge prerequisites, the researchers developed the educational module in the Arabic language including the following contents: knowledge regarding neuromuscular blockers agents (Definition neuromuscular blockers: generic and brand names, the steps of neuromuscular junction, the elements of neuromuscular control and function stability, the non-depolarizing and depolarizing neuromuscular blockers agents, therapeutic action, indications, the clinical pharmacokinetics, contraindications and cautions, adverse effects, and drug interactions).
- Knowledge regarding (Nursing considerations, nursing assessment for patients before administration, nursing diagnoses, implementation with rationale, evaluation of patients’ outcome, and clinical practice guidelines recommendation for the sustained neuromuscular blockers agents in the adult critically ill patient).
- The practical part was designed of five main aspects (Obtain and document a baseline assessment, provide sedation/analgesia as ordered, assessment parameters during administration of neuromuscular blocking agents, necessary interventions during administration of neuromuscular blocking agents, and discontinuation of administration of neuromuscular blocking agents therapy).
- The educational module material was developed using text, images and flow diagrams in Microsoft PowerPoint Presentation. The educational material was uploaded by the department of the desktop computer facility, through an internet facility on their laptops and mobile to make it accessible to the nurses and given handout. The participants could have access to the module at a time in a sequential manner. The researcher posted in the unit computer motivated the nurses to complete the module.
- The educational module was administered through combination methods that were using a traditional lecture with displaying video-based learning and doing clinical practice, which involved observational checklists to evaluate the nurses’ post-interventions. It was administered to nurses who divided into 10 groups; every group consisted of 8 nurses on the same day with the following instructions: Explain the educational module in two sessions, then keep with them for 2 weeks to read the educational module thoroughly. Nurses were allowed to ask questions in case of misunderstanding while listening and expressing interest in them.

#### II- Evaluation phase

After the module was completed, the post-intervention evaluation was completed at the end of the program. The pre- and post-intervention tests were individually monitored by the researcher. Following the implementation of the educational module, all tools, except nurses’ demographic data sheet, were ref-funded 2 weeks after receiving the educational module. Evaluate the effect of the educational module on the nurses’ knowledge and practice level to optimize the quality of care provided post-implementation monitored the adverse effects.

- Data collections and the educational module teaching for the sample of this study took about 5 months were conducted in the morning and afternoon shifts started from the middle of July 2024 until the end of December 2024.

### Administrative design

To complete the study, a letter of ethics approval was obtained from the Ethical Committee at Faculty of Nursing – Helwan University and the Recruitment Hospital Ethics Boards. Verbal informed consent has been obtained from each included nurse. Official letters were issued to them from the Faculty of Nursing – Helwan University explaining the aim of the study to obtain permission for the collection of data.

### Ethical considerations

Oral consent was taken from nurses who agree to participate in the research process after the aim of the study has been simply explained to them prior to data collection. They were assured that anonymity and confidentiality would be guaranteed and that they would have the right to withdraw from the study at any time without giving a reason. Values, culture and beliefs would be adhered to.

### Statistical design

The collected data were analyzed using (SPSS) version 24 (a total sample size of 60 nurses), to achieve a power of 95% and a level of significance of 5% (two-sided) “Dhand & Khatkar [26]”. Qualitative data were presented as number and percentage, paired with sample t-test. Correlations between different qualitative and quantitative variables were tested using correlation coefficient (person correlation). Probability (p-value) ≤ 0.05 was considered significant and < 0.001 was considered highly significant. While > 0.05 was considered non-significant.

## Results

Table 1 The percentage distribution of demographic characteristics of nurses under the study illustrated that 80% of the study nurses were females. Also, 32.5% of the study nurses were between 20 and < 25 and < 25 and 30, with a mean age of 27.50 ± 4.592 years. As regards the nurses’ educational level, 66.3% of the nurses studied had technical nursing education, while 43.3% of the nurses studied had experienced less than 5 to < 10 years, and 37.5% of them had 1 to < 5 year. Concerning the nurses attending training courses, 87.5% of nurses did not attend any training courses related to the administration of neuromuscular blocking agents.

Table 2 Percentage distribution of the satisfactory level of nurses’ knowledge regarding administration of neuromuscular blocking agents throughout pre/post phases; only (38.8, 28.7, 35.0, 37.5, 47.5, 45.0, 42.5, 46.3, 42.5 & 55.0, 58.8, & 38.8%) of the study subjects gave correct answers regarding Neuromuscular Blockers agents (definition, steps of neuromuscular junction, therapeutic action and indication, clinical pharmacokinetics, contraindications and cautions, adverse effects, drug interactions, nursing considerations, nursing assessment, diagnosis, implementation, and evaluation of patient’s outcome) at pre-implementation, compared to (86.3, 97.5, 95 & 93.8, 98.8, 93.8, 92.5, 90.0, 92.5, 97.5, and 96.3, & 90.0%) who had a satisfactory level of knowledge after educational module implementation, respectively, with a highly statistically significant difference for pre/post phases at *p* < 0.001.

Table 3 Percentage distribution of the satisfactory level of nurses’ knowledge regarding clinical practice guideline recommendations for the sustained neuromuscular blocker agents throughout pre/post phases displayed that 65.0%, 42.5%, and 40.0% of the study subjects gave correct answers regarding clinical practice guidelines (strong, weak, and good recommendations) at pre-implementation compared to 93.8%, 91.3%, and 92.5% who had a satisfactory level of knowledge after educational module implementation, respectively, with highly statistically significant differences for pre/post phases at *p* < 0.001.

Table 4 Percentage distribution of nurses’ competent level of practice regarding administration of neuromuscular blocking agents in critically ill patients throughout pre/post phases validated that, there was improvement of the studied nurses’ competent level of practice from (40.0, 37.5, 41.3, 47.5, and 56.3%) for (Obtain and document a baseline assessment, provide sedation/analgesia as ordered, assessment parameters during administration, necessary interventions, and discontinuation of neuromuscular blocking agents’ therapy) at pre-implementation compared to (92.5, 95.0, 91.3, 90.0, and 93.8%) at post-implementation phases, respectively, with highly statistically significant differences between pre/post at *P* < 0.001.

Table 5 Percentage distribution of nurses’ reported adverse effects regarding administration of neuromuscular blocking agents in critically ill patients throughout pre/post phases confirmed that there was a decrease in the incidence of nurses’ reported adverse effects from 76.3, 71.3, and 67.5 for feeling dizzy, elevation in serum potassium levels and anaphylactic reaction, arrhythmias, and hypoxia at pre-implementation to 5.0, 7.5 & 8.8, and 8.8 & 18.8 after educational module implementation, respectively, with a highly statistically significant difference for pre/post phases at *p* < 0.001.

**Fig. 1:**
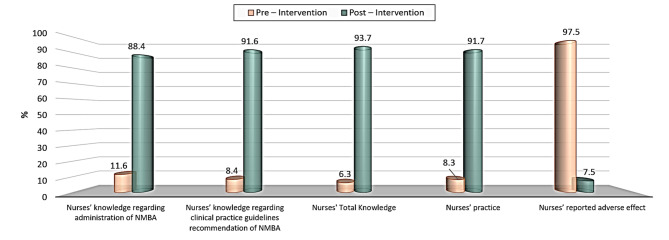
Percentage distribution regarding the total pre-post satisfactory level of knowledge and competent level of practice among study group subjects

Figure 1 Percentage distribution regarding the total pre-post satisfactory level of knowledge, competent level of practice, and reported adverse effect among study group subjects. showed that there were improvements in the studied nurses’ satisfactory level of knowledge regarding administration of NMBAs and practice guidelines, the studied nurses’ competent level of practice, and reported adverse effects from (11.6, 8.4, 6.3, 8.3, and 7.5%) at pre-implementation compared to (88.4, 91.6, 93.7, 91.7, and 97.5%) at the post-implementation phase, respectively.

Table 6 Comparison of mean scores among the study subjects regarding the total level of nurses’ knowledge and practice throughout pre/post phases clarified that there was an improvement in the studied nurses’ satisfactory level of knowledge and competent level of practice and total reported adverse effects at pre/post phases, respectively, with highly statistically significant differences between pre/post at *P* < 0.001.

Table 7 Correlation between total level of nurses’ knowledge, practice, and total reported adverse effect throughout pre/post phases revealed that there was a highly statistically insignificant difference between the total level of nurses’ knowledge, practice, and total reported adverse effect throughout pre/post phases at *p* < 0.001.

Table 8 Effect size and η2 of educational module on studied nurses’ performance regarding safe administration and adverse effects of neuromuscular blocking agents in critically ill patients throughout pre/post phases was supporting the research hypnosis and illustrated that the educational module had positive large effect size on nurses’ performance (Knowledge and practice) regarding safe administration and adverse effects of neuromuscular blocking agents in critically ill patients throughout pre/post phases of the study at η2 = 0. 729 and 0.833, as (when Eta-square value = 0.01 to < 0.06, the effect is considered weak, when it = 0.06 to < 0.14, the effect is considered medium and when it ≥ 0.14 the effect is large).

**Table 1:** The frequency and percentage distribution of demographic characteristics of nurses under the study (*n* = 60)

Item	No	%
**Gender**		
Female	64	80.0
Male	16	20.0
**Age (Years)**		
20 - < 25	26	32.5
25 - < 30	26	32.5
30 - < 35	22	27.5
35 +	6	7.5
**Mean ± SD**	**27.50 ± 4.592/ 2.10 ± 0.949**	
**Educational level**		
Diploma of nursing	22	27.5
Technical Institute of nursing	53	66.3
Bachelor of nursing	5	6.2
**Job categories**		
Staff nurse	42	52.5
Charge nurse	33	41.3
Head nurse	5	6.2
**Experience (Years)**		
1 - < 5	30	37.5
5 - < 10	35	43.8
10 +	15	18.7
**Training courses for administration of neuromuscular blocking agents**		
No	70	87.5
Yes	10	12.5

**Table 2:** Percentage distribution of the satisfactory level of nurses’ knowledge regarding administration of neuromuscular blocking agents throughout pre/post phases (no = 80)

Nurses’ Knowledge Assessment Items	Pre		Post		X2 test	
	Satisfactory		Satisfactory		Pre/post	
	no	%	no	%	X2	*P*
Definition Neuromuscular Blockers agents: Generic and Brand Names	31	38.8	69	86.3	38.507	< 0.001**
The steps of neuromuscular Junction	23	28.7	78	97.5	81.222	< 0.001**
The Four Elements of Neuromuscular Control And Function Stability	27	33.8	69	86.3	45.938	< 0.001**
The Nondepolarizing Neuromuscular Blockers agents	22	27.5	64	80.0	44.349	< 0.001**
Depolarizing Neuromuscular Blockers agents	23	28.7	68	85.0	51.601	< 0.001**
Neuromuscular Blockers agents Therapeutic Action	28	35.0	76	95.0	63.297	< 0.001**
Neuromuscular Blockers agents Indications	28	35.0	75	93.8	60.201	< 0.001**
The Neuromuscular Blockers agents clinical Pharmacokinetics	30	37.5	79	98.8	69.106	< 0.001**
The Neuromuscular Blockers agents Contraindications and Cautions	38	47.5	75	93.8	41.243	< 0.001**
Neuromuscular Blockers agents Adverse Effects	36	45.0	74	92.5	42.007	< 0.001**
Neuromuscular Blockers agents Drug interactions	34	42.5	72	90.0	40.363	< 0.001**
Nursing Considerations	37	46.3	74	92.5	40.272	< 0.001**
Nursing Assessment for patients before administration	34	42.5	78	97.5	57.619	< 0.001**
Nursing Diagnoses	44	55.0	78	97.5	39.896	< 0.001**
Implementation with Rationale	47	58.8	77	96.3	32.258	< 0.001**
Evaluation of patients’ outcome	31	38.8	72	90.0	45.812	< 0.001**

*P* < 0.01 High Significant, *P* < 0.05 Significant & *P* > 0.05 No significant

**Table 3:** Percentage distribution of the satisfactory level of nurses’ knowledge regarding clinical practice guidelines recommendation for the sustained neuromuscular blockers agents throughout pre/post phases (no = 80)

Dimensions of knowledge	Pre		Post		X2 test	
	Satisfactory		Satisfactory		Pre/post	
	no	%	no	%	X2	*P*
Strong recommendation	52	65.0	75	93.8	20.195	< 0.001**
Weak recommendation	34	42.5	73	91.3	42.913	< 0.001**
Good practice recommendation	32	40.0	74	92.5	49.308	< 0.001**

*P* < 0.01 High Significant, *P* < 0.05 Significant & *P* > 0.05 No significant

**Table 4:** Percentage distribution of nurses’ competent level of practice regarding administration of neuromuscular blocking agents in critically ill patients throughout pre/post phases (no = 80)

Competent level of practice	Pre		Post		X2 test	
	Competent		Competent		Pre/post	
	no	%	no	%	X2	*P*
Aspect 1: Obtain and document a baseline assessment	32	40.0	74	92.5	49.308	< 0.001**
Aspect 2: Provide sedation/analgesia as ordered.	30	37.5	76	95.0	59.147	< 0.001**
Aspect 3: Assessment parameters during administration of neuromuscular blocking agents	33	41.3	73	91.3	44.723	< 0.001**
Aspect 4: Necessary interventions during administration of neuromuscular blocking agents	38	47.5	72	90.0	33.629	< 0.001**
Aspect 5: Discontinuation of neuromuscular blocking agents therapy	45	56.3	75	93.8	30.000	< 0.001**

*P* < 0.01 High Significant, *P* < 0.05 Significant & *P* > 0.05 No significant

**Table 5:** Percentage distribution of nurses reported adverse effect regarding administration of neuromuscular blocking agents in critically ill patients throughout pre/post phases (no = 80)

Nurses reported adverse effect	Pre		Post		X2 test	
	occur		occur		Pre/post	
	no	%	no	%	X2	*P*
Anaphylactic reaction	57	71.3	7	8.8	65.104	< 0.001**
Anaphylactic shock	48	60.0	6	7.5	49.308	< 0.001**
Feeling dizzy	61	76.3	4	5.0	84.185	< 0.001**
Burning and pain at injection site	47	58.8	3	3.8	56.320	< 0.001**
Hypotension	53	66.3	8	10.0	53.651	< 0.001**
Bradycardia	53	66.3	12	15.0	43.556	< 0.001**
Tachycardia	51	63.7	5	6.3	58.132	< 0.001**
Arrhythmias	54	67.5	7	8.8	58.526	< 0.001**
Cardiac arrest	45	56.3	2	2.5	55.703	< 0.001**
Hyperthermia	23	28.7	2	2.5	20.907	< 0.001**
Elevation in serum potassium levels	57	71.3	6	7.5	68.100	< 0.001**
Erythema	40	50.0	2	2.5	46.618	< 0.001**
Inadequate respiratory function	49	61.3	11	13.8	38.507	< 0.001**
Bronchospasm	52	65.0	12	15.0	41.667	< 0.001**
Hypoxia	54	67.5	15	18.8	38.758	< 0.001**
Oxygen saturation decreased	53	66.3	22	27.5	24.119	< 0.001**
Muscle weakness	48	60.0	12	15.0	34.560	< 0.001**
Seizure	35	43.8	5	6.3	30.000	< 0.001**
Decreased drug effect	46	57.5	12	15.0	31.264	< 0.001**
Prolonged drug effect	36	45.0	9	11.3	22.539	< 0.001**

**Table 6:** Comparison of mean scores among the study subject regarding total level of nurses’ knowledge and practice throughout pre/post phases (no = 80)

items	Pre- test	Post- test	X2 test		
			Pre/post		
	Mean ±SD	Mean ±SD	X2	*P*	*r*
Total knowledge	45.65 ± 6.06	61.37 ± 2.01	22.007	< 0.001**	45.65 ± 6.06
Total practice	43.57 ± 5.59	57.42 ± 2.19	20.628	< 0.001**	43.57 ± 5.59
Total adverse effect	26.67 ± 2.52	36.12 ± 1.64	28.031	< 0.001**	26.67 ± 2.52

*P* < 0.01 High Significant, *P* < 0.05 Significant & *P* > 0.05 No significant

**Table 7:** Correlation between total level of nurses’ knowledge and practice throughout post phases (no = 80)

Items	Total knowledge		Total practice		Total adverse effect	
	*r*	*p*	*R*	*p*	*r*	*p*
Total knowledge			0.386	< 0.001**	0.404	< 0.001**
Total practice	0.386	< 0.001**			0.330	< 0.001**
Total adverse effect	0.404	< 0.001**	0.330	< 0.001**		

*P* < 0.01 High Significant, *P* < 0.05 Significant & *P* > 0.05 No significant

**Table 8:** Effect size and η2 of educational module on studied nurses’ performance regarding safe administration and adverse effects of neuromuscular blocking agents in critically ill patients throughout pre/post phases (*n* = 80)

Interval	Mean	SD	F Value	*P* value	η	η2	Effect size
Performance							
Pre-test	43.57	5.59	425.509	< 0.001**	0.854	0.729	0.852 Large effect
Post-test	57.42	2.19					
**Adverse effects**							
Pre-test	26.67	2.52	785.733	< 0.001**	0.912	0.833	0.911 Large effect
Post-test	36.12	1.64					

^*Significant *p* ≤ 0.05 **Highly significant *p* ≤ 0.01 F: ANOVA Test^

^* Small effect size = 0.01 to < 0.06 **Medium effect size =0.06 to < 0.14 ***Large effect size ≥ 0.14^

## Discussion

The administration of neuromuscular blocking agents (NMBAs) is a critical component of care for patients in intensive care units (ICUs), often used to facilitate mechanical ventilation, reduce oxygen consumption, and improve patient-ventilator synchrony. Despite their therapeutic benefits, NMBAs pose significant risks, including prolonged paralysis, respiratory complications, and other adverse effects if not administered correctly. The complexity of NMBA management demands a high level of knowledge and skill from nurses, who are primarily responsible for the continuous monitoring and care of critically ill patients receiving these agents. However, various studies have highlighted gaps in nurses’ understanding and adherence to safety guidelines when handling NMBAs, which can compromise patient outcomes “Lohman [27]”.

In nursing practice, educational interventions are essential for reducing difficulties associated with the administration of neuromuscular blocking agents (NMBAs). These interventions improve patient outcomes and safety by increasing confidence, knowledge, and abilities. They cover a wide range of topics, such as procedural mistakes, knowledge gaps, and the necessity of ongoing education in a subject that is changing quickly “Portela et al. [28]”.

**Regarding Gender Distribution**, the present study revealed that, most of the nurses were female. This aligns with “Attia et al., [29]” whose research titled " Intensive care nurses’ knowledge about use of neuromuscular blocking agents in patients with respiratory failure” revealed that more than half of the studied sample were female. From the researcher point of view that females dominate the nursing workforce worldwide. This could be attributed to cultural norms and societal expectations about caregiving roles. However, it’s worth noting that some countries are actively encouraging male participation in nursing, which has shown an increasing trend over recent years.

The age distribution of nurses in this study showed that nearly one-third were between 20 and 30 years old, with a mean age of 27.50 ± 4.592 years. From the researchers’ point of view this reflects a relatively young nursing workforce, which could impact both their clinical experience and adaptability to new protocols.

Similar findings were reported by Alshammari et al., [30]” whose research titled " Factors Affecting the Implementation and Barriers to Evidence-Based Practice among Nurse Practitioners in Hail Region, Saudi Arabia” reported that the majority of nurses were under 30 years, highlighting the global trend of younger professionals entering the field. However, this contrasts with findings by “Ebrahimi, Shariati & Basirinezhad [31]”, where the mean age of nurses was over 40 years, potentially due to higher career longevity and retention strategies.

**Concerning educational level**, the present study reported that about two-thirds of nurses had technical nursing education. However, it underscores the need for higher education programs to improve the competency and decision-making skills of nurses.

This is consistent with findings from “Elmaboud et al. [32]”, titled " Efficacy of educational module for nurses about “Safe care of patients under treatment of neuromuscular blockade agents” on nurses’ performance and patient’s clinical outcomes” revealed that technical nursing education was the predominant qualification among practicing nurses. Conversely, “Erbay et al., [33]”, in research titled " Medical device-related pressure injury care and prevention training program (DevICeU): Effects on intensive care nurses’ knowledge, prevention performance and point prevalence. " Revealed that in high-income countries, the proportion of nurses holding bachelor’s degrees or higher is significantly greater due to mandatory qualification policies.

Regarding years of experience, the findings of the study revealed that nearly two-fifths had 5–10 years of experience, and more than one-third had 1–5 years of experience. From the researcher’s point of view, this distribution suggests that the workforce consists largely of individuals who are still building their expertise and refining their clinical skills.

Similar results were reported by “Scanlonet al. [34]”, whose research titles " Low- and lower middle-income countries advanced practice nurses: an integrative review” found that younger nurses often dominate healthcare systems in low- to middle-income countries, as older nurses often transition to administrative roles. This may affect clinical outcomes, as experience has been linked to better clinical judgment and efficiency.

**Regarding training on neuromuscular blocking agents**, the study identified that most of nurses did not receive any training on administering neuromuscular blocking agents. From the researchers’ point of view this was due to limited institutional resources and a lack of emphasis on continuous education.

This is concerning, given the critical nature of these drugs in patient care, particularly in intensive care settings. Lohman [27]”, similarly reported in his unpublished doctoral dissertation titled " an Efficacy of a Multimedia Educational Module on Best Practices of Anesthesia Patient Safety for Neuromuscular Blockade” a lack of specialized training among nurses. In contrast, studies from high-income countries such as the USA and Canada Erin, et al., [35]”, show significantly higher training rates, driven by stringent accreditation and regulatory requirements.

**Regarding the satisfactory level of nurses’ knowledge about administration of neuromuscular blocking agents throughout pre/post phases**, the present study revealed that before the educational module, the proportion of nurses with satisfactory knowledge was notably low across various aspects of NMBA administration, such as its definition (about two-fifths), steps of the neuromuscular junction (about one-fourth), therapeutic actions and indications (about one-third), and clinical pharmacokinetics (about three-eighths). While after the educational module implementation, a remarkable improvement was observed, most of the studied nurses had satisfactory knowledge levels across most categories. This suggests that structured educational interventions can effectively address knowledge gaps, enhancing nurses’ competency in specialized and complex clinical areas like NMBA administration.

Similar findings have been reported in studies conducted from, Abouzaid et al., [36], titled with " Critical Care Nurses’ Performance Regarding Use of Neuromuscular Blocking Agents in Patients with Respiratory Failure” found that less than 40% of nurses had adequate knowledge about NMBA pharmacokinetics and indications, attributing this to a lack of formal training and reliance on on-the-job learning. In contrast, studies from developed settings, such as Erin, et al., [34], reported pre-training knowledge levels exceeding 60%, highlighting the influence of mandatory continuing education programs in enhancing baseline knowledge.

This finding aligns with the work of Lohman [27]”, demonstrated a similar impact of educational interventions where nurses’ knowledge about NMBAs increased from 40 to 92% post-training.

**Concerning the satisfactory level of nurses’ knowledge regarding clinical practice guidelines recommendation for the sustained neuromuscular blockers agents throughout pre/post phases.** The results of this study revealed that the results of this study revealed Prior to the educational intervention, less than two-thirds of the nurses studied demonstrated satisfactory knowledge of strong, weak, and good recommendations. While most of the nurses had a satisfactory knowledge improved significantly regarding all these items post program implementation.

These findings are consistent with Lohman [27], reported that nurses often lack an in-depth understanding of clinical practice guidelines due to insufficient formal training and limited access to updated protocols in hospital settings. A similar study by “Hernandez [37]”, in his study titled “Multimedia Educational Module on the Best Practices of Anesthesia Patient Safety for Quantitative Neuromuscular Monitoring” noted that only 45% of critical care nurses had adequate baseline knowledge of guideline recommendations, attributing this gap to the absence of regular continuing education programs.

**Concerning Nurses’ Competent Level of Practice Regarding Administration of Neuromuscular Blocking Agents in Critically Ill Patients.** The findings from **study** demonstrated that the competency levels of nurses regarding the administration of neuromuscular blocking agents (NMBAs) in critically ill patients showed a marked improvement following the implementation of an educational guideline. Before the intervention, nurses’ competency levels were suboptimal, ranging from approximately two-fifths to over one-half, reflecting deficiencies in practical skills, particularly in sedation and analgesia management (around two-fifths). These findings align with previous research, such as “Nemes & Renew [38]”. The study titled “Clinical practice guideline for the management of neuromuscular blockade: What are the recommendations in the USA and other countries?“, which reported that only about half of critical care nurses possessed adequate skills in safely administering NMBAs due to insufficient training and outdated protocols.

Notably, the highest pre-intervention competency level was observed in therapy discontinuation (over one-half), suggesting that nurses were more familiar with tasks commonly performed toward the end of NMBA therapy, a pattern also noted by “Park, J [39]”, in his study titled “Nursing Considerations When Using Neuromuscular Blocking Agents to Assist with Intubation”.

However, following the structured educational intervention, most of the study nurses demonstrated significant improvement in sedation and analgesia management, highlighting the crucial role of patient safety considerations during NMBA administration. From the researcher’s point of view this enhancement underscores the effectiveness of targeted training in addressing critical gaps in nursing practice. Similar improvements have been reported by “Dallı & Girgin [40]”, whose research titled “Medical device-related pressure injury care and prevention training program (DevICeU): Effects on intensive care nurses’ knowledge, prevention performance and point prevalence”. who found that structured training programs led to an 85% increase in competency for managing high-alert medications, including NMBAs and emphasize the necessity of sedation and analgesia to prevent awareness during paralysis induced by NMBAs.

**Concerning Nurses’ Reported Adverse Effects Regarding Neuromuscular Blocking Agents Administration in Critically Ill Patients.** The findings from **the present study** reveal a significant reduction in the incidence of nurses reported adverse effects associated with NMBA administration following the implementation of the educational module. These adverse effects included dizziness, elevated serum potassium levels, anaphylactic reactions, arrhythmias, and hypoxia, with pre-intervention rates significantly declining post-intervention. The observed changes were statistically significant at *p* < 0.001, from the researcher point of view this indicates the effectiveness of educational intervention.

These improvements align with findings by “Murray et al. [22]”, whose research title " Clinical practice guidelines for sustained neuromuscular blockade in the adult critically ill patient " demonstrated that structured educational programs focusing on NMBA pharmacology, adverse effect management, and monitoring significantly reduce the incidence of complications. “Fox-Duncan [41]”, corroborated this finding, whose research title " A quality improvement initiative: Educating Certified Registered Nurse Anesthetists on Reducing Residual Neuromuscular Blockage through Quantitative Monitoring” reporting that adherence to sedation guidelines minimizes the likelihood of patient discomfort and associated symptoms, such as dizziness.

**Concerning total Pre-Post Satisfactory Knowledge**,** Competent Practice Levels**,** and Reported Adverse Effects Regarding NMBAs Administration. The study results** demonstrate that substantial improvements in nurses’ knowledge, practice, and the reduction of adverse effects following the implementation of the educational module. These findings emphasize the transformative impact of structured training interventions on professional competency and patient safety in critical care settings. From the researcher’s point of view these results highlight the value of educational modules in bridging knowledge-practice gaps among nurses.

These findings are consistent with studies like “Elmaboud et al. [32]”, which reported insufficient knowledge among nurses regarding NMBA pharmacology and guidelines, often attributed to a lack of focused educational programs and clinical training opportunities. These improvements align with the findings by “Abraham [42]”, whose research titled " *Nurse education to improve use of BIS protocol for patients on neuromuscular blocking agents: a Quality initiative*” demonstrated that tailored educational interventions significantly enhance healthcare providers’ competency in administering high-risk medications, including NMBAs.

**Concerning the Mean Scores Comparison for Total Knowledge**,** Practice Levels**,** and Reported Adverse Effects** highlights significant improvements in the mean scores for nurses’ knowledge, practice competency, and reported adverse effects before and after the implementation of the educational module. From the researcher point of view these results underline the transformative role of structured education in enhancing nursing performance and reducing patient complications.

The findings agree with “Abouzaid et al. [36]”, reported that mean scores for knowledge and practice improved by over 70% following educational interventions for nurses managing critical care medications. Also, “Santos et al. [43]”, in their research titled " Effectiveness of Educational Interventions to Increase Skills in Evidence-Based Practice among Nurses: The EDITcare " observed a 60% reduction in reported adverse effects after implementing training modules focused on high-alert drug administration.

Contrasting evidence from “Ltheeth & Abbas [44]”, in their research title " Effectiveness of an Educational Program on Nurses’ Knowledge Concerning Medication Error at Teaching Hospital in AL-Nasiriyah City” suggests that while education significantly improves scores initially, maintaining high performance requires continuous reinforcement and regular assessments.

**Concerning Correlation Between Total Knowledge**,** Practice**,** and Adverse Effects**,** the result of the present study** highlights the correlation between nurses’ total knowledge, practice levels, and reported adverse effects during the pre/post phases of the study. The findings revealed a statistically insignificant difference in the correlation at *p* < 0.001 after implementing the educational module. **From the researchers` point of view** these findings emphasize that nurses require both theoretical knowledge and clinical skills to effectively prevent adverse outcomes and underscores the importance of adopting a holistic approach that integrates education with practical support and systemic enhancements.

**These results** aligned the findings of Lohman [27], reported weak correlations between knowledge and practice, highlighting that clinical environments often require skills beyond theoretical knowledge. Also, “Abouzaid et al. [36]”, emphasized that institutional changes, such as improved workflow and collaboration, significantly impact patient outcomes.

**While these results were Contrasting Findings** from “Elmaboud et al. [32]”, observed strong positive correlations between knowledge, practice, and reduced adverse effects in settings where training included robust follow-up and on-the-job supervision.

**Concerning the Effect Size and η² of Educational Module on Nurses’ Performance**,** the study results** highlight the impact of the educational module on nurses’ performance concerning the safe administration and adverse effects of neuromuscular blocking agents (NMBAs) in critically ill patients. The findings support the research hypothesis, demonstrating a large effect siz**e** (η² = 0.729 for knowledge and η² = 0.833 for practice) for the educational module’s influence on improving nurses’ performance.

From the researchers’ point of view the study results affirm the educational module’s substantial positive impact on nurses’ knowledge, practice, and safety in administering NMBAs. This underscores the value of well-designed educational interventions in enhancing nursing performance and patient outcomes.

The study results were aligned with the findings of “Mesa& Rodriguez-Diaz [45]”, in their research titled” An Educational Module on the Use of Simulation-Based Learning and Its Effects on Medication Errors and Medication Error Reporting: A Quality Improvement Project” emphasized the significant impact of structured educational modules on improving nursing practice, reporting large effect sizes (η² = 0.7–0.85) in pharmacological education. Also, “Santos et al. [43]”, observed a large effect size (η² = 0.82) in a study evaluating educational interventions on critical care nursing.

While these results were Contrasting Findings of “Michael, et al. [46]”, in their research titled “): Clinical Practice Guidelines for Sustained Neuromuscular Blockade in the Adult Critically Ill Patient” reported medium effect sizes (η² = 0.10–0.14), suggesting that limited follow-up and reinforcement can dampen the long-term impact of educational interventions.

## Conclusion

Based on the findings of the current study, it can be concluded that implementing educational modules regarding the administration of neuromuscular blocking agents is highly effective in inducing perfection of most nurses’ satisfactory level of knowledge regarding the administration of neuromuscular blocking agents, clinical practice guideline recommendations, the studied nurses’ competent level of practice, and decreasing the reported adverse effects. There are statistically highly significant differences between studied nurses’ satisfactory level of knowledge and competent level of practice and the reported adverse effect at post-implementation. Also, implementing educational modules has a large positive effect; educational module had positive large effect size on nurses’ performance (Knowledge and practice) regarding safe administration and adverse effects of neuromuscular blocking agents in critically ill patients throughout pre/post phases of the study at η2 = 0. 729 and 0.833, as (when Eta-square value = 0.01 to < 0.06, the effect is considered weak, when it = 0.06 to < 0.14, the effect is considered medium and when it ≥ 0.14 the effect is large) also, there was a highly statistically insignificant difference between the total level of nurses’ knowledge, practice, and total reported adverse effect throughout pre/post phases at *p* < 0.001. These findings emphasis on the crucial role of establishing a peer-review system where experienced nurses mentor and provide feedback to less experienced colleagues regarding NMBA-related care and creating clear, evidence-based institutional guidelines for safe NMBA use, tailored to the unique needs of critically ill patients.

### Recommendations

From the foregoing conclusion, the following recommendation is suggested:

#### Level of practice

1. Ensure all nurses working in critical care unit’s complete competency-based training on the administration and monitoring of neuromuscular blocking agents (NMBAs).
2. Introduce mandatory checklists for NMBA administration, including steps for preparation, administration, monitoring, and documentation.
3. Develop and enforce stricter protocols for continuous monitoring of neuromuscular function and vital signs post-NMBA administration.
4. Incorporate regular simulation-based scenarios to prepare nurses for managing potential complications associated with NMBAs.
5. Establish a peer-review system where experienced nurses mentor and provide feedback to less experienced colleagues regarding NMBA-related care.

#### Level of administration

1. Create clear, evidence-based institutional guidelines for safe NMBA use, tailored to the unique needs of critically ill patients.
2. Ensure adequate nurse-to-patient ratios in critical care units to facilitate safe administration and monitoring of NMBAs.
3. Provide advanced neuromuscular monitoring devices to all critical care units and train staff in their use.
4. Regularly review and audit NMBA administration practices and patient outcomes to identify areas for improvement.
5. Promote teamwork between nurses, pharmacists, and physicians to optimize NMBA use and prevent adverse effects.

#### Level of education

1. Include comprehensive modules on NMBA pharmacology, safe administration, and adverse effects in undergraduate and postgraduate nursing programs.
2. Conduct regular workshops and seminars to update nurses on the latest advancements in NMBA safety and administration.
3. Develop online educational modules, including interactive case studies and quizzes, for convenient access to NMBA-related training.
4. Motivate nurses to pursue certifications in critical care nursing to strengthen their skills in managing critically ill patients requiring NMBAs.
5. Make evidence-based guidelines, textbooks, and journal articles on NMBA management readily available to nursing staff.

#### Level of further research

1. Conduct longitudinal studies to evaluate the sustained effectiveness of educational modules on NMBA-related nursing performance.
2. Investigate organizational, personal, and technological barriers that hinder safe NMBA administration.
3. Assess the relationship between nurses’ participation in educational modules and patient outcomes, such as reduced adverse effects.
4. Study the effectiveness of simulation, virtual reality, and AI-based tools in improving NMBA-related nursing competencies.
5. Research the impact of interdisciplinary training modules involving nurses, pharmacists, and physicians on NMBA safety.

## Electronic supplementary material

Below is the link to the electronic supplementary material.

Supplementary Material 1

## Data Availability

No datasets were generated or analysed during the current study.
