# Supplementary material for: Effectiveness of an educational module on nurses’ performance regarding safe administration and adverse effects of neuromuscular blocking agents in critically ill patients
Source: BMC Nurs. 2025 Jul 22;24:963. doi: 10.1186/s12912-025-03600-0 (PMC12281960; doi:10.1186/s12912-025-03600-0)
Supplement: Supplementary file 1 — Supplementary Material 1 [file 12912_2025_3600_MOESM1_ESM.pdf]

Assessment® Questionnaire for Educational Module on Nurses' Performance Regarding Safe Administration and Adverse Effects of Neuromuscular Blocking Agents in Critically Ill Patients

Code No. ( )

Dear Nurses, the aim from this group of questions is to assess effectiveness of educational module on nurses' performance regarding safe administration and adverse effects of neuromuscular blocking agents in critically ill patients, every member has the right to share in this study or not share without explain causes, all data present in this questionnaire are secret and will used only in scientific research only and thank you.

1- Agreement to share in the study.
1. Yes
2. No

2- Name (Optional): ……………………………………….

3- Sex:
1. Male
2. Female

4- Age:
1. 25 - < 30 year
2. 30 - < 35 year
3. 35 + year

5- Level of Education:
1. Nursing Secondary School
2. Technical Nursing Institute
3. Baccalaureate degree of nursing

6- Position
1. Nurse
2. Charge Nurse
3. Head Nurse

7- Intensive care unit: ( )

8- Experience years

1. 1- < 5 year
2. 5 – < 10 year
3. 10 + year

9- Do you have previous training courses for administration of neuromuscular blocking agents

1. Yes
2. No

Nurses Knowledge Assessment Questionnaire Regarding administration of neuromuscular blocking agents

The items on this sheet were adapted from **Adeyinka & Layer (2024) and Iavarone et al. (2024).**

Code No. ( )

Dear participant, please choose your responses using the following choice:

No.	Assessment items	Know	Unknown
1.	Definition Neuromuscular Blockers agents: Generic and Brand Names		
2.	The 7 steps of neuromuscular Junction		
3.	The Four Elements Of Neuromuscular Control And Function Stability		
4.	The Nondepolarizing Neuromuscular Blockers agents		
5.	Depolarizing Neuromuscular Blockers agents		
6.	Neuromuscular Blockers agents Therapeutic Action		
7.	Neuromuscular Blockers agents Indications		
8.	The Neuromuscular Blockers agents clinical Pharmacokinetics		
9.	The Neuromuscular Blockers agents Contraindications and Cautions		
10.	Neuromuscular Blockers agents Adverse Effects		
11.	Neuromuscular Blockers agents Drug interactions		
12.	Nursing Considerations		
13.	Nursing Assessment for patient before administration		
14.	Nursing Diagnoses		
15.	Implementation with Rationale		
16.	Evaluation of patients outcome		
	**Total**		

Nurses knowledge regarding clinical practice guidelines for the sustained neuromuscular blockade in the adult critically ill patient

The items on this sheet were adopted from **Murray et al. (2016)**

Code No. ( )

Assessment Items	Know	Unknown
**Clinical practice recommendation (s)**		
**Strong recommendation**		
1. Scheduled eye care with lubrication and eyelid closure		
**Weak recommendation**		
1. Continuous infusion of NMBA rather than intermittent boluses		
2. Avoid use in status asthmatics		
3. Trial of NMBA in life-threatening situations with hypoxemia, respiratory acidosis, and hemodynamic compromise		
4. May be used to manage overt shivering in therapeutic hypothermia		
5. PNS with inclusive clinical assessment may be a useful tool for determining the depth of blockade		
6. PNS should not be used alone (without clinical assessments) in patients receiving a continuous infusion of NMBAs		
7. Implementation of a structured physiotherapy regimen		
8. Target blood glucose level < 180 mg/dL		
9. Dose NMBA based on ideal body weight or		

adjusted boy weight (rather than actual)		
**Good practice recommendation**		
1. PNS can be used with clinical assessment in patients undergoing therapeutic hypothermia		
2. Protocols should be utilized to guide NMBA administration in patients undergoing therapeutic hypothermia		
3. Analgesic and sedative drugs should be used before and during neuromuscular blockade		
4. Implement measures to reduce risk of unintended extubation in patients receiving NMBAs		
5. Reduce dosing in patients with myasthenia gravis based on PNS use		
6. Discontinue NMBAs prior to determining brain death		
**Total**		

Nurses' practical observational checklists

It were adopted from **(Bittner, 2023 and Wiegand, 2017)** to evaluate the nurses' practical competency level throughout administration of neuromuscular blocking agents in critically ill patients

Code No. ( )

Items	Competent	In Competent
**1. Obtain and document a Baseline Assessment**		
1- Vital Signs		
2- Hemodynamic status (B/P, HR and pulmonary artery pressures if available)		
3- Ventilatory status (mode of ventilation, rate, FiO2, peak inspiratory pressures and SaO2. ETCO2 may also be monitored and documented)		
4- Neurological status (LOC, mental status, pupillary response and movement of extremities).		
5- General skin integrity assessment.		
6- Comfort/pain status. If patient conscious, utilize Wong-Baker Scale. If unconscious, use changes in vital signs, and observation of tearing, grimacing and/or diaphoresis and FLACC pain scale.		
7- Location of electrodes and condition of skin. for correct use of Peripheral Nerve Stimulator (PNS))		
8- Amount of current (milliamps) required to elicit the supramaximal stimulation (SMS), or four vigorous twitches. **(Perform after maximal analgesia and sedation, but before administration of (NMBA)**		
**2. Provide Sedation/analgesia as ordered.**		
**3. Assessment Parameters During Neuromuscular Blockade:**		
1. Obtain vital signs every 1 hour.		
2. Hemodynamic monitoring parameters routinely.		
3. Ventilatory status with careful attention to monitoring PIP (positive inspiratory pressure) and SaO2.		
4. Neuro assessment every 1 hour (LOC, mental status, pupillary response and movement of extremities).		

5. Adequacy of analgesia/sedation every 1 hour.		
6. Monitor changes in VS, tearing, diaphoresis forpain assessment (Note: FLACC scale no longer applicable).		
7. Electrolytes, BUN, creatinine and liver function tests as ordered.		
8. Train of Four (TOF) approximately 15 minutes after the bolus dose. Continue to retest every 1-2 hours until clinically stable and a satisfactory level of blockade is achieved. Once an adequate level is achieved, re-test every 4-8 hours.		
**4. Necessary Interventions During Neuromuscular Blockade:**		
1- Administer sedation/analgesia per physician orders, to assure adequate levels.		
2- Change body position every 2 hours or more often as needed. Consider a pulmonary treatment bed.		
3- Skin integrity assessments every 2 hours with turning.		
4- Apply skin care devices as ordered.		
5- Apply lubricants/artificial tears every 1 hour to prevent conjunctival and scleral injury.		
6- Place a sign above the patient's bed stating, "patient is pharmacologically paralyzed".		
7- Reorient the patient every 1 hour.		
8- Explain all procedures and nursing care.		
9- For patients pharmacologically paralyzed for prolonged periods, consider a Physical Therapy consult if not contraindicated.		
**5. Discontinuation of NMBA therapy:**		
1- Continually re-assess the need for NMBA. Discontinue as needed to assess status of patient.		
2- Neuro assessments to include LOC, mental status, pupillary response, movement of extremities and motor strength every 1 hour until patient has returned to baseline.		
3- Ventilatory status		
4- Scleral and conjunctival care every 4 hours Assess patient for need of restraints according to Hospital Policy.		
